# Chinese experience on comparison of clinical efficacy and safety of hemodialysis and peritoneal dialysis in the treatment of diabetic kidney failure: a systematic review and meta-analysis

**DOI:** 10.3389/fmed.2023.1116103

**Published:** 2023-08-09

**Authors:** Zhifeng Wei, Yujie Jin, Jinxiu Cheng, Xiaoli Han, Junfen Liu, Shengjun Liu

**Affiliations:** Department of Nephrology, The Frist Affiliated Hospital of Hebei North University, Zhangjiakou, Hebei, China

**Keywords:** hemodialysis, peritoneal dialysis, kidney failure, safety, efficacy

## Abstract

**Objective:**

This meta-analysis aims to compare the efficacy and safety of peritoneal dialysis (PD) and hemodialysis (HD) in the treatment of diabetic kidney failure.

**Methods:**

Five databases were selected to retrieve research on PD and HD for diabetic kidney failure until 6 August 2022. A fixed-effects or random-effects model was utilized to calculate the standardized mean difference (SMD) or odds ratio (OR) based on the heterogeneity among studies.

**Results:**

Sixteen studies were included. The results showed that patients with diabetic kidney failure treated with PD had lower levels of albumin, total protein, and systolic blood pressure (SBP) and higher levels of urine volume, creatinine, and blood urea nitrogen (BUN) and lower risk of cardiovascular and bleeding events, with significant statistical difference when compared with patients treated with HD (albumin: SMD = −1.22, 95%CI: −1.53, −0.91; total protein: SMD = −0.96, 95%CI: −1.16, −0.77; SBP: SMD = −0.35, 95%CI: −0.64, −0.06; urine volume: SMD = 0.68, 95%CI: 0.40, 0.96; creatinine: SMD = 0.49, 95%CI: 0.27, 0.72; BUN: SMD = 0.55, 95%CI: 0.25, 0.85; cardiovascular events: OR = 0.42, 95%CI: 0.28, 0.62; bleeding: OR = 0.41, 95%CI 0.27, 0.62).

**Conclusion:**

This meta-analysis summarized the advantages and disadvantages of PD and HD for treating diabetic kidney failure patients. Compared with HD, PD is more effective in preserving residual kidney function, reducing hemodynamic effect, and lowering the risk of bleeding and cardiovascular events in diabetic kidney failure patients, but it also predisposes to protein-energy malnutrition and increases the risk of infection.

## Introduction

Diabetic kidney disease is the irreversible change of kidney structure and function, which 30%–40% of diabetic patients will develop, primarily manifested as proteinuria (1, 2). As diabetic kidney disease worsens, it will gradually evolve into kidney failure which is defined by the glomerular filtration rate declining to 15 mL/min/1.73 m^2^ or less and starting dialysis treatment (3, 4). There might be no obvious symptoms in the early stage of kidney failure. However, toxins accumulate in the body following the gradual decline of kidney function which can lead to various symptoms of uremia, such as acidosis, electrolyte metabolism disorder, cardiac insufficiency, digestive system symptoms, and anemia (5, 6). Many metabolic wastes cannot be customarily excreted from the body because of severe impairment of kidney function in kidney failure patients. With the accumulation of toxic substances in the body, various organs and tissues will appear to different degrees of damage, and organ failure and death could occur in severe cases. Hence, timely and effective removal of toxic substances in kidney failure patients is currently the fundamental method for treating and relieving symptoms of kidney failure patients (7, 8).

At present, treatment of diabetic kidney failure patients includes dialysis and kidney transplantation (9). Dialysis includes peritoneal dialysis (PD) and hemodialysis (HD). The principle of HD is to use a semi-permeable membrane and concentration gradient to exchange dialysate and blood substance in the dialyzer which could achieve the removal of toxins and maintain the electrolyte balance effect. However, the puncture of the fistula required for each dialysis session can significantly impact the Vivo environment and hemodynamics, leading to faster loss of residual kidney function and a greater chance of infection (10, 11). PD could efficiently remove the retained metabolites, correct the balance of electrolytes and acid–base, and remove excess body fluids in the kidney failure patient’s body by using the semi-permeability characteristics of the patient’s peritoneum and the principle of diffusion and convection to exchange solutes between blood and dialysate which could effectively preserve the patient’s residual kidney function (12, 13). Compared with HD, PD has the following advantages of less impact on the cardiovascular system and hemodynamics, providing better protection of residual kidney function, performing easily at home, and removing macromolecules more effectively (14). However, compared with HD, studies have found that PD has a higher incidence rate of peritonitis. It may be related to the volume overload status of kidney failure patients after PD (15). PD-related peritonitis would accelerate protein loss to energy-protein malnutrition in diabetic kidney failure patients, aggravate kidney impairment, and lead to peritoneal sclerosis with a reduced effect of PD (16). A meta-analysis of retrospective studies also showed that HD might significantly benefit survival in diabetic kidney failure patients compared to PD (17).

The widespread application of PD in treating patients with diabetic kidney failure has shown promising efficacy and safety. While limited studies which were mostly conducted in Korea and incomprehensive content related to the clinical practice of PD and HD treatment for diabetic kidney failure patients were summarized (18). This review aims to accomplish a comprehensive evaluation of the clinical efficacy and safety of PD vs. HD in the treatment of diabetic kidney failure patients and serve as a supplemental meta-analysis of studies in this field accomplished outside Korea (18). It could contribute to implementing and innovating the treatment method for diabetic kidney failure patients.

## Methods

This systematic review and meta-analysis referred to the guidance to authors in the Cochrane Handbook for Systematic Reviews of Interventions Version 6.3 (updated February 2022) (19). This systematic review and meta-analysis is registered at the International Prospective Register of Systematic Reviews (Number CRD42022349799).

### Search strategy

Five electronic databases including Pubmed, Web of Science, Cochrane Library, China National Knowledge Infrastructure, and WANFANG DATA were systematically searched for randomized controlled trials (RCTs) comparing PD and HD for treating diabetic kidney failure patients up to 6 August 2022, by two researchers (W ZF and C JX), to provide a comprehensive evaluation of both clinical efficacy and safety in treating patients with diabetic kidney failure. The search keywords were “peritoneal dialysis,” “hemodialysis,” “diabetes,” “kidney failure,” and “end-stage renal disease.” The detail of the search strategy was listed in Supplementary Table 1. In addition, we also manually scanned the references of retrieval studies and related systematic reviews to maximize the recall rate of associated literature to avoid the omission of relevant articles in the electronic database search.

### Inclusion and exclusion criteria

The selection process was conducted by two independent researchers (J YJ and C JX). Title, abstract and full text of retrieved studies were evaluated strictly in accordance with the inclusion and exclusion criteria. A third researcher (W ZF) was consulted in disputed studies between the two researchers to make a final decision on the inclusion or exclusion of the study in the quantitative synthesis. The inclusion criteria were presented as participants, interventions, comparisons, outcomes, and study design (PICOS) protocol. Participants: the study participants were all patients with diabetic kidney failure. Intervention and comparison: the treatment method for HD and PD were included in the study, and the clinical efficacy and safety of the two treatment methods were compared in treating patients with diabetic kidney failure. Outcomes: the study included, but was not limited to, clinical signs, laboratory tests, and patient complications. Study design: only RCTs were included for ensuring good quality of combined results. Exclusion criteria (all studies that met the following conditions were excluded): (1) The full text was unavailable. (2) Literature such as reviews, conferences, lectures, case reports, and abstracts that we cannot obtain the data required for meta-analysis were excluded. (3) The same trial was repeatedly published. (4) The study design had significant flaws, or the results were reported with significant bias.

### Data extraction

Two researchers (J YJ and C JX) extracted the data listed below from each study that met inclusion criteria by using a pre-designed data extraction table to establish the database. It contained basic information about the included study such as journal, title, first author, year of publication, and baseline characteristics of the study population like the number of diabetic kidney failure patients included in the study, grouping, age, duration of treatment, and indicator related to clinical efficacy and safety which cover clinical signs before and after treatment, laboratory indicators, complications. In addition, the inclusion and exclusion criteria of the study population, and indicators related to the study design were documented. A third researcher (W ZF) would crosscheck the data after completing the data extraction for data consistency.

### Quality assessment

Two independent researchers (J YJ and C JX) used the risk of bias assessment section of the Cochrane Handbook which is the most widely used and valid tool after multiple verifications for the quality assessment of RCTs. Research meeting the criteria was included in the quantitative synthesis (19). The evaluation criteria were based on selection bias, performance bias, detection bias, attrition bias, reporting bias, and other biases which are the most important study design issues for RCTs. If there was an argument concerning the quality of included studies, the third researcher (W ZF) was inquired for determining the degree of risk bias. In the last, the overall quality assessments were rated as “Low risk,” “High risk,” or “Unclear,” and were shown by the risk of bias plots.

### Statistical analysis

Results were pooled across studies with STATA version 15.1 (Stata Corp MP., College Station, TX, United States) (20, 21). All the included patients were diabetic kidney failure patients who were divided into PD and HD treatment groups randomly, patients had good clinical consistency. The heterogeneity across the included studies was measured by using the Q test (Chi-square test) and the *I^2^* test. *p*-value and *I^2^* value were combined to determine the heterogeneity and homogeneity among studies. If *p* > 0.05, homogeneity was considered among studies in the Q test. Otherwise, it was heterogeneity. *I^2^* values of 0%–39%, 40%–59%, and ≥60% were considered low, medium, and high heterogeneity between studies, respectively (19). A fixed-effects model was utilized to combine effect size when there was homogeneity and low heterogeneity in *I^2^* value among studies. Otherwise, a random-effects model was utilized to combine results when there was heterogeneity and medium or high heterogeneity in *I^2^* value among studies. The random-effects analysis was labeled at the bottom of the forest plot, while the fixed-effects analysis was blanked. The standardized mean difference (SMD) and its 95% confidence interval (CI) were calculated for continuous variables to compare the clinical efficacy and safety of the two treatment methods in patients with diabetic kidney failure. The odds ratio (OR) and the 95%CI were compared for dichotomous variables. A forest plot was used to show the meta-analysis results for each indicator. Egger linear regression was utilized to assess the publication bias of the result. Besides, the sensitivity of the pooled effect size was evaluated by Duval and Tweedie’s trim and fill test (22, 23). If the pooled effect size occurred a significant change after being trimmed and filled, it indicated the meta-analysis result was unstable. *p* < 0.05 was considered statistically significant.

## Results

### Literature search, study characteristics, and quality assessment

**Figure 1:**
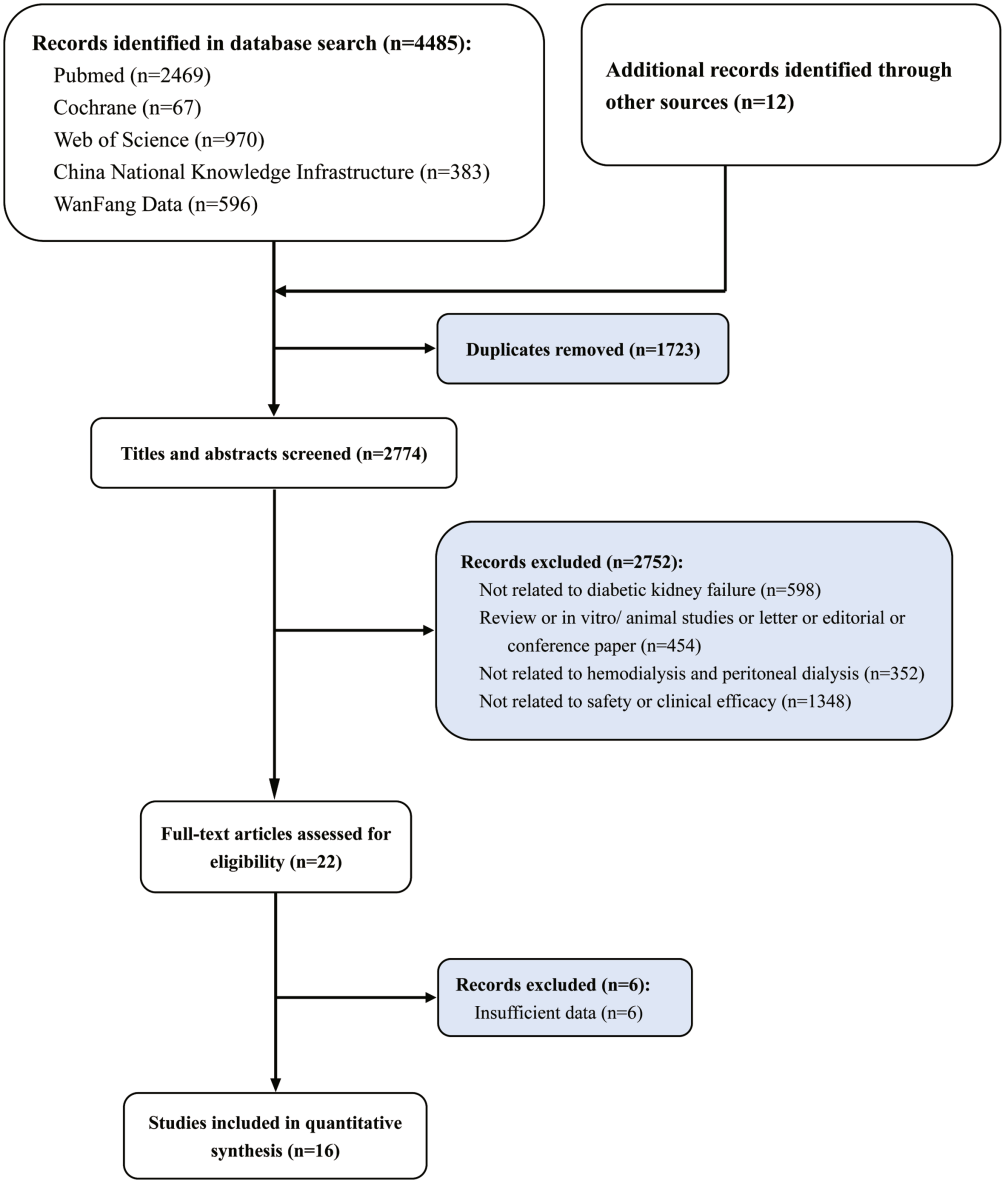
Study selection flowchart, systematic review and meta-analysis of comparison of clinical efficacy and safety of hemodialysis and peritoneal dialysis in the treatment of diabetic kidney failure.

A total of 4,485 articles were obtained by searching five databases, and 12 articles were obtained from the manual search of references of initial inclusion in the literature. After removing 1,723 repeatedly retrieved articles, 2,752 articles did not meet the inclusion criteria (not related to diabetic kidney failure *n* = 598; review or *in vitro*/animal studies or letter or editorial or conference paper *n* = 454; not related to hemodialysis and peritoneal dialysis *n* = 352; not related to clinical efficacy or safety *n* = 1,348). Subsequently, six studies were excluded from the full-text evaluation process because they were not available or translated into valid data. Finally, 16 studies were included in the meta-analysis (Figure 1). A total of 635 diabetic kidney failure patients treated with PD and 719 diabetic kidney failure patients treated with HD were included in this meta-analysis. The baseline characteristics of the 16 RCT studies included in the meta-analysis are shown in Table 1 (24–39).

**Table 1:** Baseline characteristics of included studies for meta-analysis.

References	Number of cases		Age (y, PD/HD)	Course of treatment (mo)	Indicators
	PD	HD			
Zhao et al. (24)	17	19	55.3 ± 9.7/54.3 ± 9.6	3	Weight, urine volume, SBP, DBP, serum creatinine, BUN, cholesterol, triglycerides, total protein, albumin, hemoglobin, phosphorus, calcium, glucose, complications
Guo et al. (25)	21	21	52.4 ± 5.7/51.7 ± 5.2	6	SBP, DBP, serum creatinine, BUN, total protein, albumin, hemoglobin, glucose, cholesterol, triglycerides, complications
Song et al. (26)	28	32	63.3 ± 8.1/62.1 ± 7.6	3	SBP, DBP, total protein, albumin
Li et al. (27)	42	73	62.1 ± 12.5/60.9 ± 10.3	46 (median)	SBP, DBP, albumin, hemoglobin, phosphorus, calcium, complications
Li et al. (28)	43	55	62.6 ± 10.9/60.4 ± 11.3	36	Weight, urine volume, SBP, DBP, serum creatinine, BUN, total protein, albumin, hemoglobin, glucose, complications
Tang et al. (29)	19	21	54.0 ± 9.6/53.1 ± 9.3	≥ 3	Urine volume, SBP, DBP, serum creatinine, BUN, cholesterol, triglycerides, total protein, albumin, hemoglobin, phosphorus, glucose
Zhao et al. (30)	30	30	62.4 ± 7.6/60.8 ± 6.9	12	Urine volume, SBP, DBP, serum creatinine, BUN, cholesterol, triglycerides, albumin, hemoglobin, complications
Yuan and Wang (31)	51	76	57.1 ± 10.9/57.4 ± 10.6	12	Weight, urine volume, SBP, DBP, serum creatinine, BUN, cholesterol, triglycerides, total protein, albumin, hemoglobin, phosphorus, calcium, glucose, complications
Peng et al. (32)	60	60	40.4 ± 5.6/40.7 ± 5.8	≤ 36 OR > 36	Albumin, glucose, cholesterol, triglycerides
Yi et al. (33)	40	40	65.7 ± 1.3/66.1 ± 1.3	6	SBP, DBP, albumin, hemoglobin
Ma et al. (34)	50	50	52.4 ± 13.7/52.8 ± 13.5	44 (median)	SBP, DBP, albumin, hemoglobin, phosphorus, calcium
Shi and Zhou (35)	41	44	57.2 ± 10.4/57.4 ± 10.5	12	Serum creatinine, BUN, complications
Huang and Ma (36)	50	50	56.7 ± 7.9/56.9 ± 7.9	12	Urine volume, SBP, DBP, serum creatinine, BUN, total protein, albumin, hemoglobin, phosphorus, calcium, complications
Duan et al. (37)	43	55	63.3 ± 8.7/60.9 ± 7.3	12	Weight, urine volume, SBP, DBP, serum creatinine, BUN, cholesterol, triglycerides, total protein, albumin, hemoglobin, phosphorus, calcium, glucose, complications
Ji et al. (38)	60	53	53.6 ± 3.7/52.4 ± 3.9	12	SBP, DBP, serum creatinine, BUN, glucose complications
Fei et al. (39)	40	40	62.8 ± 10.7/60.9 ± 10.2	6, 12	Calcium, phosphorus, urine volume

PD, Peritoneal dialysis; HD, hemodialysis; y, year; mo, months; SBP, systolic blood pressure; DBP, diastolic blood pressure; BUN, blood urea nitrogen.

**Figure 2:**
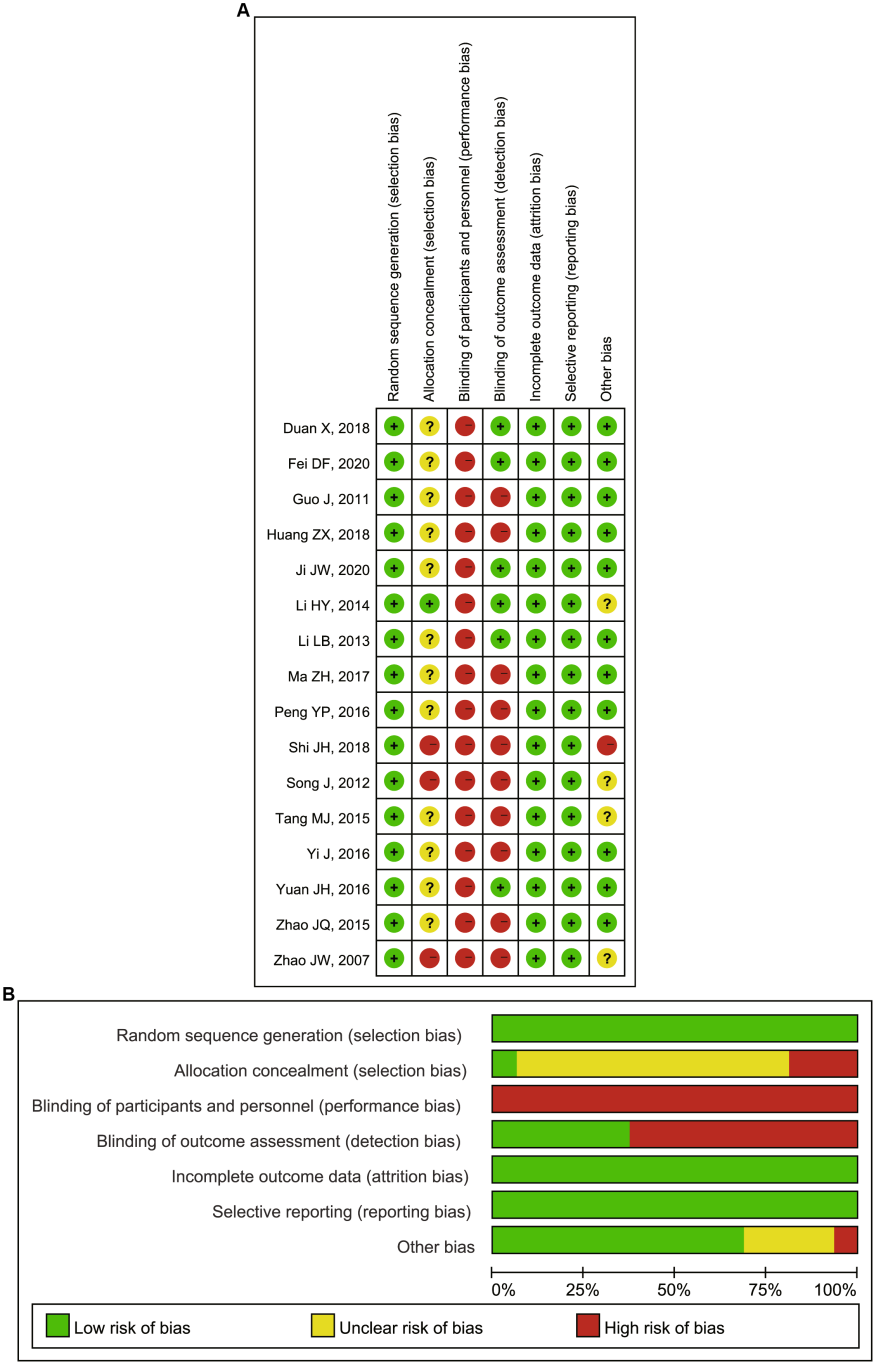
**(A)** Risk of bias summary: review authors’ judgments about each risk of bias item for each included RCTs; **(B)** Risk of bias graph: review authors’ judgments about each risk of bias item presented as percentages across all included RCTs. RCTs, randomized controlled trials.

All the included articles were systematically assessed for their bias, such as selection bias, performance bias, detection bias, reporting bias, and attrition bias according to the quality assessment section of the Cochrane Handbook. Notably, prior to the study, each study excluded patients with the following conditions: (1) acute cardiovascular or cerebrovascular events within 2 months, (2) clinically detectable acute or chronic infections, (3) patients with a malignant tumor, severe hepatic impairment, active immune system disease and hormone or immunosuppressants usage, and (4) a history of trauma or surgery within 1 month. Therefore, most of the studies had no significant missing data that would have compromised the power of the test but a limited extrapolation of the results. Both biases were rated as low risk of bias. But blinding of participants and personnel and blinding of outcome assessment were uncontrollable in this field. Almost all performance bias and detection bias in this Meta-analysis were rated as high risk. In addition to this, the overall assessment of the remaining included RCTs was considered a low risk of bias, indicating the good quality of this meta-analysis and the high reliability of the results (Figures 2A,B).

### Indicators

#### Comparison between PD and HD for diabetic kidney failure

**Figure 3:**
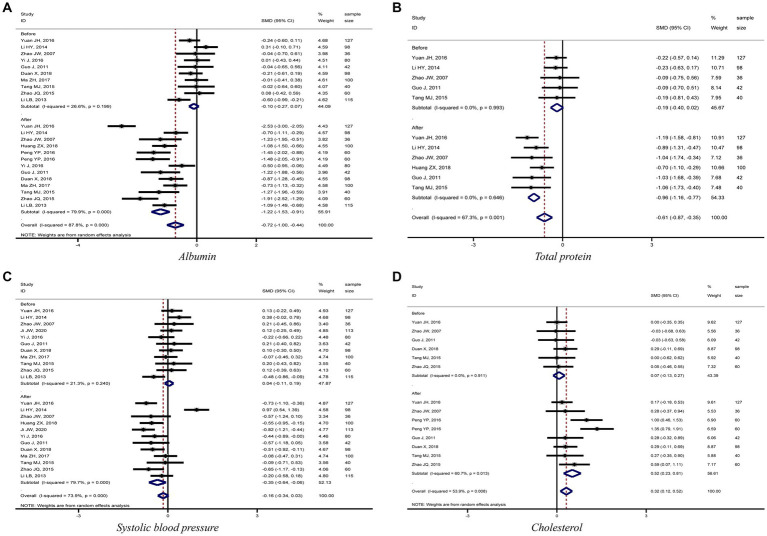
Forest plot of comparison between PD and HD for diabetic kidney failure: **(A)** albumin; **(B)** total protein; **(C)** systolic blood pressure; **(D)** cholesterol. HD, hemodialysis; PD, Peritoneal dialysis.

**Figure 4:**
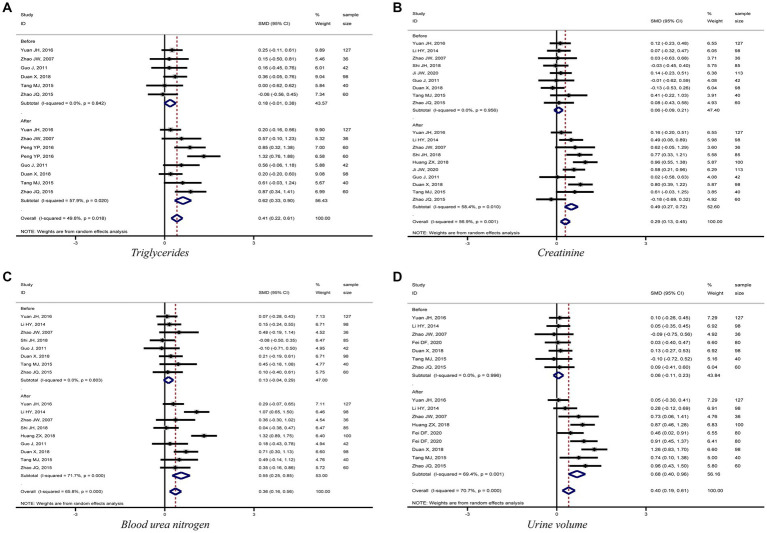
Forest plot of comparison between PD and HD for diabetic kidney failure: **(A)**, triglycerides; **(B)**, creatinine; **(C)**, blood urea nitrogen; **(D)**, urine volume. HD, hemodialysis; PD, Peritoneal dialysis.

The meta-analysis results showed that diabetic kidney failure patients treated with PD had lower levels of albumin, total protein (TP), and systolic blood pressure (SBP) compared to diabetic kidney failure patients treated with HD, with statistically significant differences (albumin: SMD = −1.22, 95%CI: −1.53, −0.91; total protein: SMD = −0.96, 95%CI: −1.16, −0.77; SBP: SMD = −0.35, 95%CI: −0.64, −0.06; Figures 3A–C). In addition, patients treated with PD showed higher levels of cholesterol, triglycerides, creatinine, BUN, and urine volume than those treated with HD (cholesterol: SMD = 0.52, 95%CI: 0.23, 0.81; triglycerides: SMD = 0.62, 95%CI: 0.33, 0.90; creatinine: SMD = 0.49, 95%CI: 0.27, 0.72; BUN: SMD = 0.55, 95%CI: 0.25, 0.85; urine volume: SMD = 0.68, 95%CI: 0.40, 0.96; Figures 3D, 4A–D). The remaining clinical and laboratory indicators such as diastolic blood pressure, weight, calcium, phosphorus, hemoglobin, and glucose did not differ statistically significantly after treatment with PD or HD in patients with diabetic kidney failure (Supplementary Figures 1, 2).

**Figure 5:**
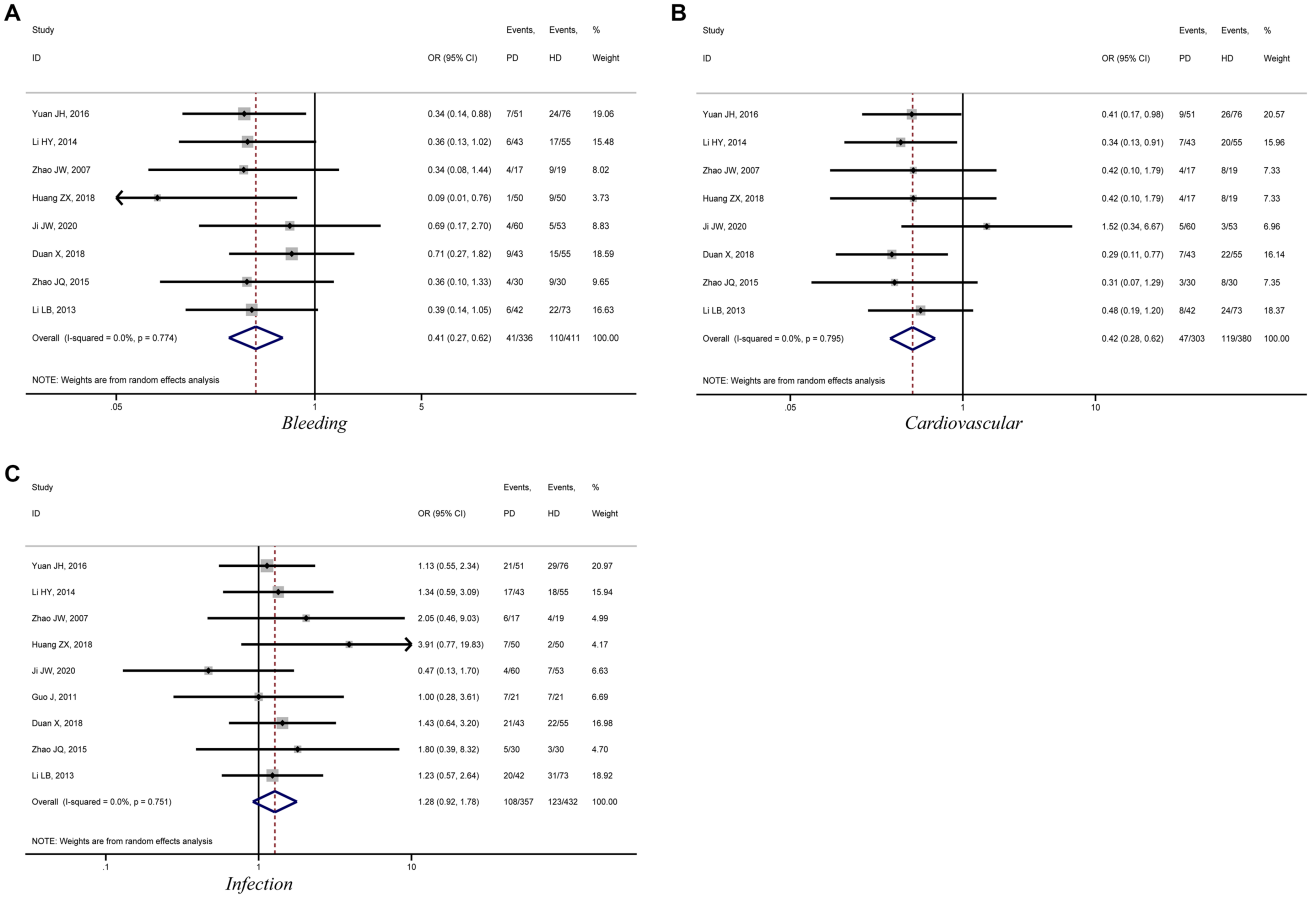
Forest plot of comparison between PD and HD for diabetic kidney failure: **(A)**, bleeding; **(B)**, cardiovascular; **(C)**, infection. HD, hemodialysis; PD, Peritoneal dialysis.

This meta-analysis analyzed the incidence of bleeding, infection, and cardiovascular events in patients with diabetic kidney failure treated with PD and HD. Eight studies compared the incidence of bleeding and cardiovascular events in patients treated with PD and HD. The meta-analysis results showed that patients treated with PD had a lower risk of bleeding and cardiovascular events compared to those treated with HD (bleeding: OR = 0.41, 95%CI: 0.27, 0.62; cardiovascular: OR = 0.42, 95%CI: 0.28, 0.62; Figures 5A,B). However, the risk of infection was higher among patients treated with PD than among those treated with HD, but the difference was not statistically significant (OR = 1.28, 95%CI: 0.92, 1.78; Figure 5C).

#### Publication bias assessment and sensitivity analysis

The publication bias of each indicator related to the efficacy and safety of the two kinds of dialysis treatment was analyzed using Egger linear regression. We did not observe significant publication bias among the indicators, indicating results were not affected significantly by small sample size studies. In addition, Duval and Tweedie’s trim and fill sensitivity test did not generate trim and fill in the indicators of this meta-analysis, suggesting that the effect size of each indicator was stable in this meta-analysis (Supplementary Table 2).

## Discussion

Patients with diabetic kidney failure often have various complications such as glucose metabolism disorders, hypertension, cardiovascular and cerebrovascular diseases, infection and malnutrition, and important factors like preserving residual kidney function which should be paid more attention to in kidney replacement treatment. PD and HD, the most commonly used kidney replacement therapies, can replace part of the kidney function and improve patients’ prognoses. It is very important to compare the two dialysis treatments for the control of complications in patients with kidney failure. In contrast to HD, PD has advantages and shortcomings in the treatment process for patients with diabetic kidney failure. The latest meta-analysis conducted in 2019 shows that HD may have an advantage over PD for the survival of diabetic kidney failure patients (17). However, the advantages and disadvantages of the two dialysis methods for patients with diabetic kidney failure were absent. Our meta-analysis included 16 RCTs to evaluate the efficacy and safety of PD and HD in treating patients with diabetic kidney failure, and to show their advantages and disadvantages from each other.

This meta-analysis showed that PD patients had lower blood albumin and total protein levels than HD-treated diabetic kidney failure patients. The forest plots, Figures 3A,B, demonstrated that kidney failure patients treated with PD had lower blood albumin and total protein levels than those treated with HD in each study and the differences were all statistically significant. Egger’s test also concluded that both indicators had no significant publication bias in the meta-analysis results. The Duval and Tweedie’s trim and fill test for both indicators did not generate trim and fill, indicating that the effect sizes for both indicators were stable. PD has a high ultrafiltration rate, which could lead to excessive loss of proteins, amino acids, and peptides. It might be associated with lower protein levels in diabetic kidney failure patients treated with PD than in patients treated with HD. It is related to the fact that patients treated with PD are prone to protein-energy malnutrition, microinflammation, decreased immunity, and an increased chance of infection due to decreased albumin levels (Figure 5C) (40–42).

PD presents a better role in lowering SBP and protecting residual kidney function reflected by maintaining urine volume in diabetic kidney failure patients when compared with HD. Results demonstrated that patients treated with PD had lower levels of SBP and higher levels of urine volume, BUN, and creatinine than patients treated with HD, and the differences were statistically significant. Furthermore, the meta-analysis results for the four indicators were stable and without significant publication bias. It is mainly related to the fact that PD uses the peritoneum as a dialysis membrane to remove medium and macromolecular toxins (e.g., vasopressor substances) from the body by diffusion and ultrafiltration. In addition, PD has less impact on human hemodynamics and kidney blood flow, which can ensure kidney perfusion to protect residual kidney function better (43, 44). To sum up, this meta-analysis fully demonstrated the advantages of PD in treating diabetic kidney failure patients.

Calcium and phosphorus metabolism disorders are common in maintenance dialysis patients, with the main clinical manifestations being metastatic calcification, osteodystrophy, and hypercalcemia. Studies have found that long-term calcium and phosphorus metabolism disorders in maintenance dialysis patients can increase the risk of adverse cardiovascular events, which is a significant cause of death (17, 45). Hypercalcemia could exacerbate vascular calcification and hyperphosphatemia is a risk factor for coronary artery calcification in patients with diabetic kidney failure. The results of this meta-analysis showed that the two treatments were equivalent in reducing patients’ blood calcium and phosphorus levels. Moreover, the original research included in the meta-analysis presented that both treatments can significantly reduce blood calcium and phosphorus levels in patients. They suggested that both treatments can effectively remove substances such as blood calcium and phosphorus, thus contributing to the improvement of calcium and phosphorus metabolic disorders in patients (27, 29, 31, 34, 36, 37, 39).

This meta-analysis found that diabetic kidney failure patients treated with HD had a higher incidence of bleeding and cardiovascular events than PD. The usage of anticoagulants, operation of dialysis access puncture and extracorporeal circulation, poor hemodynamics control, and susceptibility to microangiopathy damages during HD were responsible for these complications. However, patients treated with PD need to be vigilant about aseptic procedures to reduce the risk of infection.

### Limitations

The main limitations of this meta-analysis are as follows. First, the studies included in this meta-analysis were mainly focused on the Chinese region. Therefore, whether the findings of this study can be extrapolated to other countries needs to be confirmed by more studies in the future. Second, the follow-up time of some of the RCTs was too short to assess the long-term effects of both dialysis methods on diabetic kidney failure patients. Therefore, more RCTs with large samples of diabetic kidney failure patients around the world and long follow-up periods were needed to confirm the advantages and disadvantages of PD or HD in treating diabetic kidney failure patients. Meanwhile, new studies should be integrated with existing results of the meta-analysis for comparison and to reinforce the long-term effect and regional applicability of meta-analysis results.

## Conclusion

This meta-analysis summarized the advantages and disadvantages of PD and HD in treating diabetic kidney failure patients. First, PD is effective in preserving residual kidney function in diabetic kidney failure patients and reducing hemodynamic effects, but it also predisposes to adverse effects such as protein-energy malnutrition and an increased risk of infection. In contrast, HD can significantly improve patients’ hypoproteinemia but also increases the risk of bleeding and cardiovascular events. Therefore, factors such as the advantages and disadvantages of both dialysis methods, patients’ specific conditions, and long-term prognosis should be considered comprehensively to improve patients’ health and reduce the incidence of adverse events.

## Data availability statement

The original contributions presented in the study are included in the article/Supplementary material, further inquiries can be directed to the corresponding author.

## Author contributions

ZW, YJ, and SL: critical revision of the manuscript. ZW, YJ, JC, and SL: substantial contribution to the conception and design of the work. ZW, JC, XH, and JL: manuscript drafting and acquisition, analysis, and interpretation of the data. ZW, YJ, JC, XH, JL, and SL: revising the manuscript critically. All authors contributed to the article and approved the submitted version.

## Funding

This paper was supported by the Zhangjiakou City Science and Technology Research and Development Plan (grant no. 1911021D-6).

## Conflict of interest

The authors declare that the research was conducted in the absence of any commercial or financial relationships that could be construed as a potential conflict of interest.

## Publisher’s note

All claims expressed in this article are solely those of the authors and do not necessarily represent those of their affiliated organizations, or those of the publisher, the editors and the reviewers. Any product that may be evaluated in this article, or claim that may be made by its manufacturer, is not guaranteed or endorsed by the publisher.

## Supplementary material

Supplementary Figure 1 Forest plot of comparison between PD and HD for diabetic kidney failure: **(A)** diastolic blood pressure; **(B)** weight; **(C)** calcium; **(D)** phosphorus. HD: hemodialysis; PD: Peritoneal dialysis.

SUPPLEMENTARY FIGURE 2 Forest plot of comparison between PD and HD for diabetic kidney failure: **(A)** hemoglobin; **(B)** glucose. HD: hemodialysis; PD: Peritoneal dialysis.

SUPPLEMENTARY TABLE 1 The detail of search strategy in meta-analysis.

SUPPLEMENTARY TABLE 2 Evaluation of publication bias and sensitivity analysis.

## Abbreviations

HD: Hemodialysis
PD: Peritoneal dialysis
RCTs: Randomized controlled trials
SMD: Standardized mean difference
CI: Confidence interval
ORs: Odds ratios
SD: Standard deviation
SBP: Systolic blood pressure
TP: Total protein
