# Supplementary figures and images for: Chinese experience on comparison of clinical efficacy and safety of hemodialysis and peritoneal dialysis in the treatment of diabetic kidney failure: a systematic review and meta-analysis
Source: Front Med (Lausanne). 2023 Aug 9;10:1116103. doi: 10.3389/fmed.2023.1116103 (PMC10449255; doi:10.3389/fmed.2023.1116103)

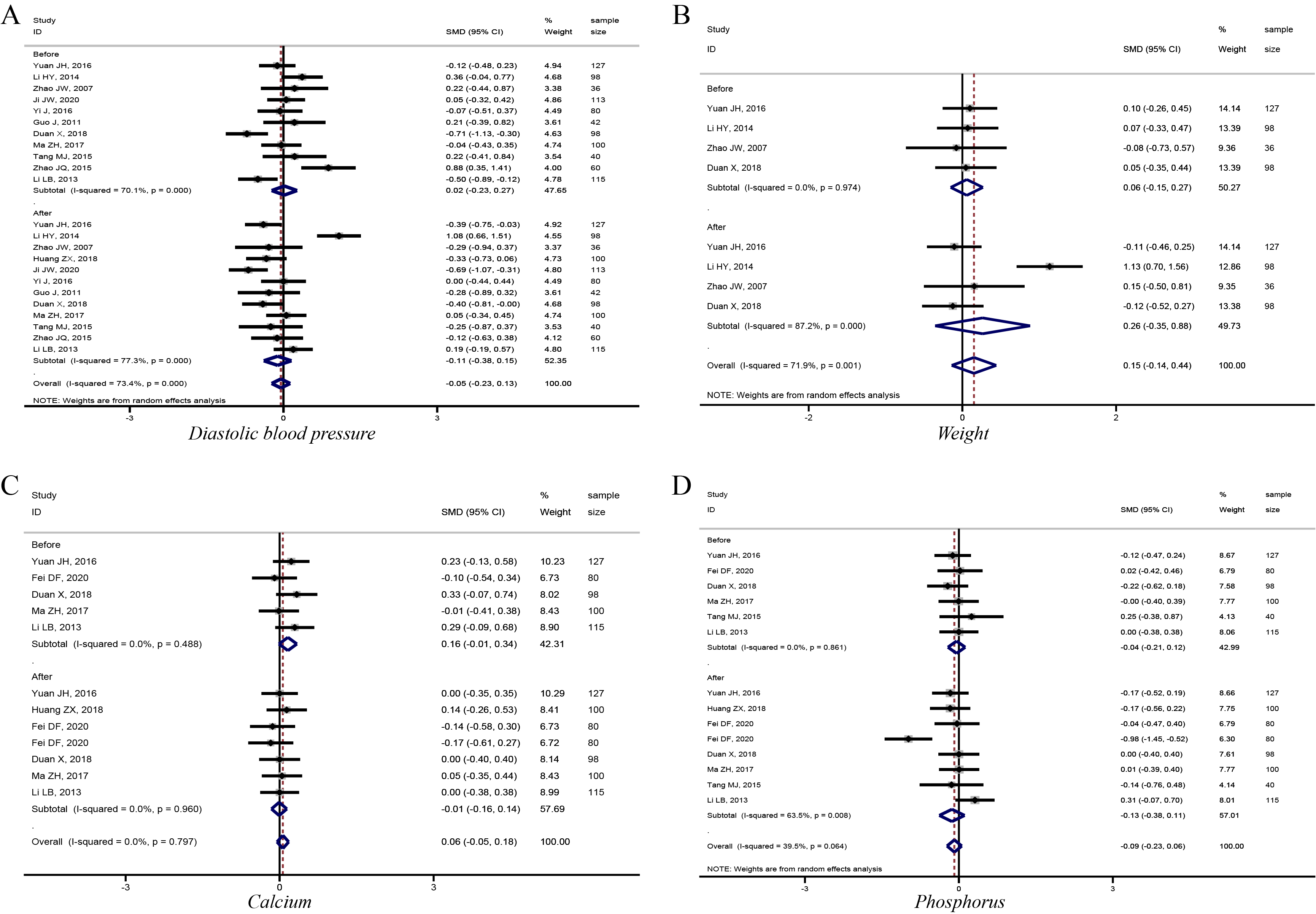

Supplement: Supplementary Figure 1 — Forest plot of comparison between PD and HD for diabetic kidney failure: (A) diastolic blood pressure; (B) weight; (C) calcium; (D) phosphorus. HD: hemodialysis; PD: Peritoneal dialysis. [file Image_1.PNG]

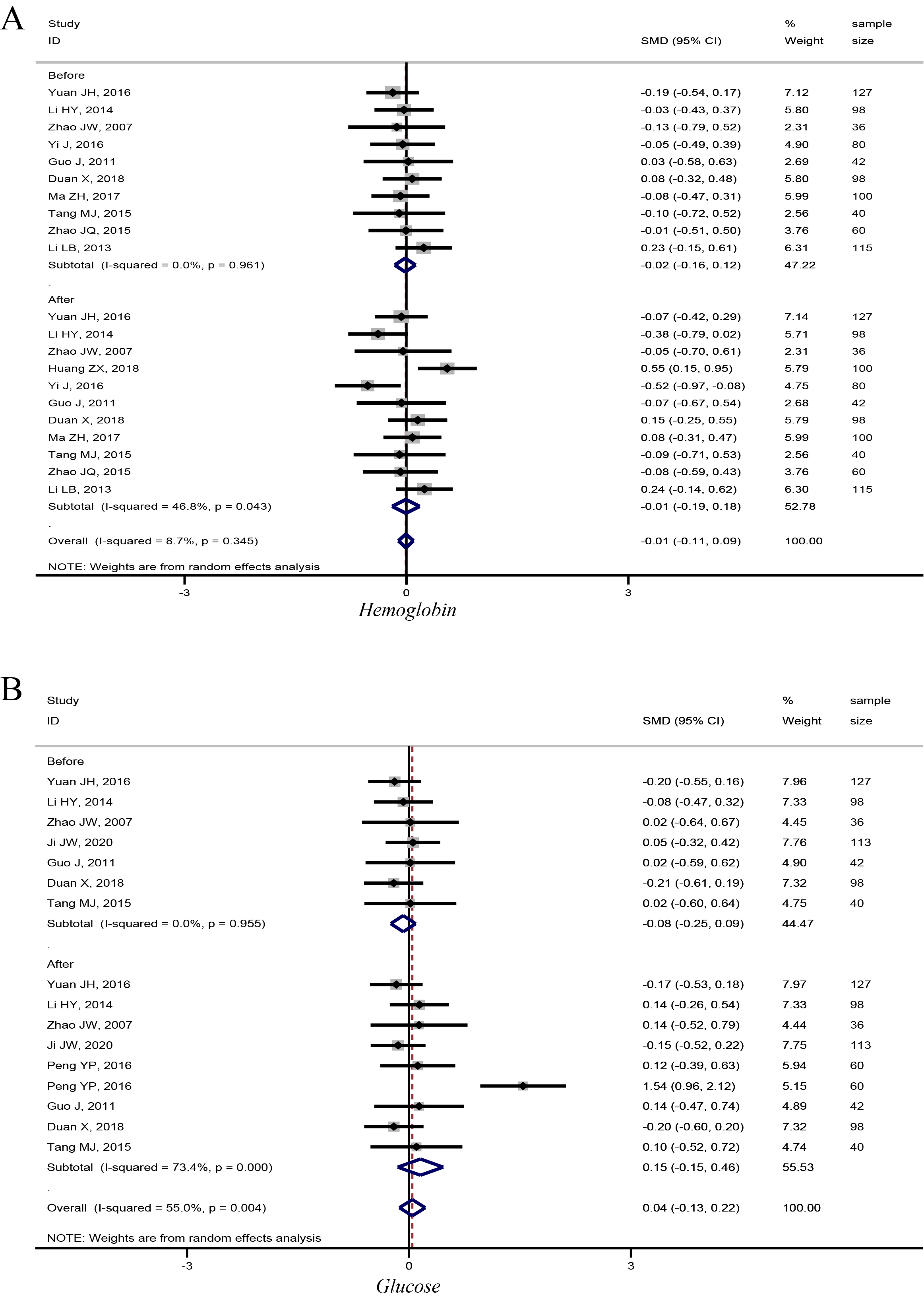

Supplement: SUPPLEMENTARY FIGURE 2 — Forest plot of comparison between PD and HD for diabetic kidney failure: (A) hemoglobin; (B) glucose. HD: hemodialysis; PD: Peritoneal dialysis. [file Image_2.PNG]
