# Supplementary material for: Chinese experience on comparison of clinical efficacy and safety of hemodialysis and peritoneal dialysis in the treatment of diabetic kidney failure: a systematic review and meta-analysis
Source: Front Med (Lausanne). 2023 Aug 9;10:1116103. doi: 10.3389/fmed.2023.1116103 (PMC10449255; doi:10.3389/fmed.2023.1116103)
Supplement: SUPPLEMENTARY TABLE 1 — The detail of search strategy in meta-analysis. [file Table_1.DOCX]

**Supplemental Table 1. The detail of search strategy in Meta-analysis.**

| **Pubmed** |
| --- |
| ((peritoneal dialysis) OR (peritoneal dialysis[MeSH Terms])) AND ((hemodialysis) OR (hemodialysis[MeSH Terms])) AND ((diabetes) OR (diabetes[MeSH Terms])) AND ((end-stage kidney disease) OR (end-stage kidney disease[MeSH Terms]) OR (kidney failure) OR (kidney failure[MeSH Terms])) |
| **Web of science** |
| TS=(peritoneal dialysis) AND TS=(hemodialysis) AND TS=(diabetes) AND (TS=(end-stage kidney disease) OR TS=(kidney failure)) |
| **Cochrane** |
| (peritoneal dialysis) AND (hemodialysis) AND (diabetes) AND ((end-stage kidney disease) OR (kidney failure)) Searching was conducted in TITLE, ABSTRACT and KEYWORDS. |
| **China National Knowledge Infrastructure** |
| Subject terms: "peritoneal dialysis," "hemodialysis," "diabetes," "kidney failure," and "end-stage kidney disease" were chosen to search in TITLE, ABSTRACT and KEYWORDS in the same Boolean logic of pubmed. |
| **WAN FANG DATA** |
| Subject terms: "peritoneal dialysis," "hemodialysis," "diabetes," "kidney failure," and "end-stage kidney disease" were chosen to search in TITLE, ABSTRACT and KEYWORDS in the same Boolean logic of pubmed. |
