# Supplementary material for: Chinese experience on comparison of clinical efficacy and safety of hemodialysis and peritoneal dialysis in the treatment of diabetic kidney failure: a systematic review and meta-analysis
Source: Front Med (Lausanne). 2023 Aug 9;10:1116103. doi: 10.3389/fmed.2023.1116103 (PMC10449255; doi:10.3389/fmed.2023.1116103)
Supplement: SUPPLEMENTARY TABLE 2 — Evaluation of publication bias and sensitivity analysis. [file Table_2.DOC]

**Supplemental table 2. Evaluation of publication bias and sensitivity analysis.**

| Index | Egger’s regression | |  | Duval and Tweedie’s trim and fill | | |
| --- | --- | --- | --- | --- | --- | --- |
|  | Intercept | *p* |  | Original effect size | Studies trimmed | Adjusted effect size |
| Albumin | -4.146 | 0.162 |  | -0.72 (-1.00, -0.44) | 0 | -0.72 (-1.00, -0.44) |
| Bleeding | -4.097 | 0.421 |  | 0.41 (0.27, 0.62) | 0 | 0.44 (0.04, 0.85) |
| Calcium | -5.783 | 0.179 |  | 0.06 (-0.05, 0.18) | 0 | 0.06 (-0.05, 0.18) |
| Cardiovascular | 0.995 | 0.304 |  | 0.42 (0.28, 0.62) | 0 | 0.42 (0.28, 0.62) |
| Cholesterol | 1.362 | 0.461 |  | 0.32 (0.12, 0.52) | 0 | 0.32 (0.12, 0.52) |
| Creatinine | -0.149 | 0.933 |  | 0.29 (0.13, 0.45) | 0 | 0.29 (0.13, 0.45) |
| DBP | 2.089 | 0.330 |  | -0.05 (-0.23, 0.13) | 0 | -0.05 (-0.23, 0.13) |
| Glucose | 2.941 | 0.170 |  | 0.04 (-0.13, 0.22) | 0 | 0.04 (-0.13, 0.22) |
| Hemoglobin | -0.883 | 0.463 |  | -0.01 (-0.11, 0.09) | 0 | -0.01 (-0.11, 0.09) |
| Infection | 1.970 | 0.189 |  | 1.28 (0.92, 1.78) | 0 | 1.28 (0.92, 1.78) |
| Nitrogen | 0.149 | 0.944 |  | 0.36 (0.16, 0.56) | 0 | 0.36 (0.16, 0.56) |
| Phosphorus | -0.649 | 0.787 |  | -0.09 (-0.23, -0.06) | 0 | -0.09 (-0.23, -0.06) |
| SBP | 0.554 | 0.801 |  | -0.16 (-0.34, 0.03) | 0 | -0.16 (-0.34, 0.03) |
| Total protein | -0.187 | 0.935 |  | -0.61 (-0.87, -0.35) | 0 | -0.61 (-0.87, -0.35) |
| Triacylglycerol | 1.904 | 0.270 |  | 0.41 (0.22, 0.61) | 0 | 0.41 (0.22, 0.61) |
| Urine volume | 1.803 | 0.463 |  | 0.40 (0.19, 0.61) | 0 | 0.40 (0.19, 0.61) |
| Weight | 0.862 | 0.815 |  | 0.15 (-0.14, 0.44) | 0 | 0.15 (-0.14, 0.44) |

Abbreviation: SBP = systolic blood pressure, DBP = diastolic blood pressure.
